# Variations in the quality of malaria-specific antibodies with transmission intensity in a seasonal malaria transmission area of Northern Ghana

**DOI:** 10.1371/journal.pone.0185303

**Published:** 2017-09-25

**Authors:** Kwadwo A. Kusi, Emmanuel A. Manu, Theresa Manful Gwira, Eric Kyei-Baafour, Emmanuel K. Dickson, Jones A. Amponsah, Edmond J. Remarque, Bart W. Faber, Clemens H. M. Kocken, Daniel Dodoo, Ben A. Gyan, Gordon A. Awandare, Frank Atuguba, Abraham R. Oduro, Kwadwo A. Koram

**Affiliations:** 1 Department of Immunology, Noguchi Memorial Institute for Medical Research, College of Health Sciences, University of Ghana, Legon, Ghana; 2 Department of Biochemistry, Cell and Molecular Biology, College of Basic and Applied Sciences, University of Ghana, Legon, Ghana; 3 West African Centre for Cell Biology of Infectious Pathogens, College of Basic and Applied Sciences, University of Ghana, Legon, Ghana; 4 Department of Parasitology, Biomedical Primate Research Centre, Rijswijk, The Netherlands; 5 Navrongo Health Research Centre, Ghana Health Service, Navrongo, Ghana; 6 Department of Epidemiology, Noguchi Memorial Institute for Medical Research, College of Health Sciences, University of Ghana, Legon, Ghana; Universidade Federal de Minas Gerais, BRAZIL

## Abstract

**Introduction:**

*Plasmodium falciparum* induced antibodies are key components of anti-malarial immunity in malaria endemic areas, but their antigen targets can be polymorphic. Induction of a high proportion of strain-specific antibodies will limit the recognition of a broad diversity of parasite strains by these responses. There are indications that circulating parasite diversity varies with malaria transmission intensity, and this may affect the specificity of elicited anti-malarial antibodies. This study therefore assessed the effect of varying malaria transmission patterns on the specificity of elicited antibody responses and to identify possible antibody correlates of naturally acquired immunity to malaria in children in an area of Ghana with seasonal malaria transmission.

**Methods:**

This retrospective study utilized plasma samples collected longitudinally at six time points from children aged one to five years. Multiplex assays were used to measure antibody levels against four *P*. *falciparum* AMA 1 variants (from the 3D7, FVO, HB3 and CAMP parasite strains) and the 3D7 variant of the EBA 175 region II antigen and the levels compared between symptomatic and asymptomatic children. The relative proportions of cross-reactive and strain-specific antibodies against the four AMA 1 variants per sampling time point were assessed by Bland-Altman plots. The levels of antibodies against allelic AMA1 variants, measured by singleplex and multiplex luminex assays, were also compared.

**Results:**

The data show that increased transmission intensity is associated with higher levels of cross-reactive antibody responses, most likely a result of a greater proportion of multiple parasite clone infections during the high transmission period. Anti-AMA1 antibodies were however associated with a history of infection rather than protection in this age group.

**Conclusion:**

The data contribute to understanding the underlying mechanism of the acquisition of strain-transcending antibody immunity following repeated exposure to diverse parasite strains.

## Introduction

The World Health Organization [1] reports that there has been a steady decline in the global incidence and deaths due to malaria over the last 15 years. The disease however continues to be of considerable public health importance due to the detrimental impact and burden it places on many countries, especially those in sub-Saharan Africa. Children greatly suffer from malaria since naturally acquired immunity to the clinical forms of the disease develops slowly and is usually not sterile [2]. Immune responses to a number of parasite antigens have been shown to be targets of immune responses but the specific antigens that mediate protection from infection and clinical disease have not as yet been fully described.

Antibodies have been demonstrated to play an important role in the partial protection against clinical malaria [3–5]. High levels of antibodies against blood stage parasite antigens such as apical membrane antigen 1 (AMA-1), erythrocyte binding antigen (EBA-175), the merozoite surface proteins (MSPs), reticulocyte-binding protein homologue (Rh5), Glutamate-rich protein (GLURP) and circumsporozoite protein (CSP) have either singly or collectively been associated with a reduced risk of clinical malaria in various malaria endemic populations [6–9]. Indeed, data from a number of high throughput antibody analysis studies indicate that antibody-mediated protective immunity is more likely to be associated with responses to a wide diversity of antigen targets rather than single antigen targets [7,8,10,11]. Despite the importance of having high levels of these antibodies in order to attain a semi-immune status, the quality of these antibodies is also very important as that defines the functionality of elicited antibodies. Most of the parasite antigens that have currently been identified as targets of immune responses do exhibit some level of polymorphism, and this affects the functional quality of previously elicited antibody responses [2,12,13]. Antibody-mediated immunity against *P*. *falciparum* has been shown in both experimental animal models and human challenge studies to be parasite strain-specific since functional responses elicited against a particular parasite strain do not yield comparable levels of inhibition against heterologous strains [14–17]. This suggests that the observed antibody-mediated immunity at least has a component that is strain-specific [18,19]. On this basis, the attainment of clinical immunity against malaria will depend on the acquisition of various antibody specificities following exposure to multiple parasite variants over time [20,21].

Studies in naturally infected humans however show that repeated exposure to malaria infection could potentially lead to a broadening of antibody specificity [14,22–24]. It has also been demonstrated that repeated exposure to different parasite strains subsequently leads to the predominant induction or boosting of antibodies that recognize epitopes that are shared by the various parasite strains [2,25]. Thus clinical immunity against malaria may also be mediated by cross-reactive or strain-transcending antibody responses, and this has clearly been demonstrated *in vitro* using antibodies from animals that were immunized with combinations of known polymorphic antigens [26–28].

An assessment of the relevance of strain-specific and cross-reactive responses to clinical malaria immunity in naturally exposed individuals is however complex since the parasite exposure history in such individuals is usually unknown and the cross-reactive or strain-specific description is dependent on this history. We have however attempted an analysis of the relative proportions of cross-reactive and strain-specific antibodies to a set of polymorphic antigens in cross-sectional plasma samples from naturally exposed individuals [23]. In the current study, we apply this approach to assess the acquisition of antigen-specific cross-reactive and strain-specific antibodies against malaria parasites using samples collected in a longitudinal study involving children between the ages of one and five years.

## Methods

### Ethical statement

This study made use of archived human plasma samples which were obtained from a longitudinal cohort study conducted between 2004 and 2005 in the Kassena-Nankana District of the Upper East region of Ghana. The original study was approved by the Ghana Ministry of Health and ethical clearance was sought from the Ethics Committees of the Navrongo Health Research Centre (FWA00000250) and Noguchi Memorial Institute for Medical Research, Legon (FWAA00001824). Informed consent was obtained from parents of the participating children for the original study, and the current analyses of archived samples were done under renewed approvals from the two ethical bodies mentioned above.

### Study site and sample information

The study site (Kassena-Nankana District) is a savanna region with subsistence farming being the main occupation of the inhabitants. The region has had two main seasons; at the time the study was conducted, there was a dry season that spanned between November and April the following year, and a rainy season which was between May and October [29,30]. Malaria transmission is seasonal and overlaps with the rain patterns. The malaria attack rate in the district during the study period was approximately 3.5 attacks per child per year [31]. Exclusion criteria for the study included, amongst others, children presenting with common chronic diseases, those with other acute febrile illness and anaemic children with haemoglobin < 6 g/dl of blood at the time of recruitment. Participants who were diagnosed as having uncomplicated malaria during the study were treated with either chloroquine or sulphadoxine-pyrimethamine (Fansidar®) as the first-line drugs according to Ghana Ministry of Health policy at the time. Additional detailed description of the study site and population have been published previously [30].

For the original study that collected plasma samples, a cohort of 325 children aged 1 to 5 years were enrolled at the end of the low malaria transmission season (May) in 2004 and were followed up over a one year period (till May 2005) that spans the high and low transmission seasons. At the beginning of the study, and at two-month intervals, 0.5–1.0 ml of finger-prick blood samples were collected from the participants whose parents had consented to enroll their children in the study. A total of seven samples per child were taken over the one year period and these were processed and stored for laboratory analysis. At each sampling time point, body temperature measurements were taken and parasite infection status was determined by microscopic examination of blood smears and by malaria RDTs, while haemoglobin levels were measured by Hemocue. Demographic information and data relating to the subjects’ malaria exposure during the transmission season was captured by a study questionnaire. Children who were febrile (axillary temperature ≥ 37.5°C) and tested positive for malaria parasites were referred to the nearest designated health facility for appropriate treatment.

### Study subject categorization

For the current study, archived plasma samples from six of the seven contact time points (excluding the baseline samples) were retrieved from storage for analysis. Samples were grouped into two categories based on the clinical and parasitological data from the original study. The first group, described as the symptomatic (***Symp***) group, included children who had one or more clinical malaria episodes. Clinical malaria was defined as having blood film parasitaemia, a fever (temperature ≥ 37.5°C) and at least one other common malaria symptom at any time during the one year study period and no other obvious cause for the fever. The second category was the asymptomatic (***Asymp***) group, which included children who had blood film parasitaemia but with no fever and no other clinical symptom of malaria. In total, samples from 126 children (six samples per child, making a total of 756 plasma samples) were selected for analysis. Sixty-four (64) of the 126 children were in the symptomatic study group and 62 were in the asymptomatic group.

### Antigens

The full length ectodomain (amino acids 25–545) of the FVO (Genbank accession number AJ2642667) variant of AMA1 was expressed as a recombinant protein in *Pichia pastoris* under good manufacturing practice (GMP) conditions and purified by a methodology that has been previously described [32]. The three other variant AMA1 antigens, HB3 (GenBank: U33277), 3D7 (GenBank: U65407) and CAMP (GenBank: M58545) were produced under similar conditions and purified using a slight modification of the methodology described for three *in silico* designed AMA1 Diversity Covering (DiCo) proteins [33]. The hydrophobic interaction purification step utilized butylsepharose FF on a BioRad purifier, rather than Butyl 650M Toyopearl in the reference above. All antigens were devoid of N-glycosylation sites [16] and were recognized by the reduction-sensitive rat monoclonal antibody 4G2, suggesting a correct folding of proteins. Region II of the EBA-175 antigen from the 3D7 parasite variant (EBA-175 RII, GenBank accession number AAB51672) was also expressed in *P*. *pastoris* as a non-glycosylated antigen under GMP conditions.

### Coupling of antigens to microspheres

The four AMA1 variant antigens and the EBA-175 RII antigen were coupled to microspheres or beads (Luminex Inc., Austin., TX, USA) with unique spectral addresses (101, 102, 103, 104 and 105) for the measurement of antigen-specific antibody levels by the Luminex xMAP technology. Bovine serum albumin (BSA) was coupled to a sixth bead set (106) for the assessment of non-specific binding in all assays.

The coupling reaction was performed as previously described [30]. Briefly, each of the stock bead suspensions was repeatedly vortexed and sonicated, and 200 μl aliquots were dispensed into labelled microcentrifuge tubes (Eppendorf) and centrifuged at 310 x g for 5 minutes. Beads were afterwards washed twice with 80 μl of 0.1 M sodium dihydrogen phosphate buffer (pH 6.2) and pelleted by centrifugation at 11,300 x g for 2 minutes. Washed beads were re-suspended in 80 μl of activation buffer and dispersed by repeated gentle sonication and vortexing. Carboxyl functional groups on the surface of beads were then activated by the addition of 10 μl of a 50 mg/ml solution of 1-ethyl 3-(3-dimethylamino-propyl) carbodiimide hydrochloride (EDC), followed immediately by 10 μl of a 50 mg/ml solution of sulfo-N-hyroxysulfosuccinimide (Sulfo NHS). The tube contents were gently mixed and incubated at room temperature in the dark for 20 minutes. Activated beads were washed twice with 250 μl of 0.05M of 2-(N-morpholine) ethanesulfonic acid (MES, pH 5.0) and subsequently re-suspended in 100 μl of the same buffer. Antigens that had been diluted to the appropriate predetermined concentrations (10 μg/ml for the AMA1 variants, 25 μg/ml for EBA-175 RII and 100 μg/ml for BSA) in 400 μl of the MES buffer were then added to the activated beads, mixed by repeated vortexing and sonication and incubated at room temperature in the dark for 2 hours with rotational mixing. After this period, coupled beads were pelleted and washed, once with 1 ml of PBS pH 7.4, containing 0.05% Tween 20, and twice with 1 ml PBS, 1% BSA, pH 7.4, containing 0.05% sodium azide. Coupled beads were finally re-suspended in 1 ml PBS, 1% BSA, pH 7.4, containing 0.05% sodium azide. The bead concentration for coupled antigens was determined by light microscopy using a haemocytometer and the beads stored at 4°C in the dark until use.

Protocol for antigen coupling and for performance of the multiplex assay has been deposited in protocols.io (https://www.protocols.io/private/587a8b11e41cf045b36ffa515bb5a632).

### Multiplexed measurement of antigen-specific antibody levels

Plasma antigen-specific antibodies were measured using multiplex assays on the Luminex 200 x-MAP platform (Luminex Inc., Austin, TX USA). Plasma samples from the 126 children as well as pools of positive and negative control plasma samples were assayed for antibodies specific to the four AMA1 allelic variants and the EBA 175RII antigen. Plasma samples were diluted 1:400 for test and negative control plasma samples, and 1:10,000 for positive control pool. Antigen-specific coupled beads were pooled, mixed and 50 μl aliquots added to wells in a washed Multiscreen filter base plate (Millipore, Billerica, MA). Diluted plasma samples were subsequently added to duplicate wells and the plate vortexed and incubated at 4°C in the dark for one hour. After this period, the plate was washed three times and incubated with 25 μl/well of 5 μg/ml of biotin-labelled goat anti-human detection antibody in the dark at 4°C for 1 hour. This step was followed by incubation with 1:50 dilution of streptavidin-conjugated phycoerythrin (25 μl/well) for 30 minutes at 4°C in the dark. The plate was subsequently developed by addition of 25 μl/well of 0.5% formaldehyde in PBS and read on a programmed Luminex 200 system that runs on the xPonent 3.1 software (Luminex Corporation). Results were expressed as mean fluorescent intensity (MFI) and this is directly proportional to the antigen-specific antibody concentration. Bovine serum antigen (BSA) was included in all assays to account for nonspecific antibody binding by subtracting the average BSA antibody level for each plate from the antibody level of test wells before statistical analysis was performed.

### Comparison of multiplex and singleplex assays for measuring antibodies to polymorphic antigens

The feasibility of measuring the levels of antibodies against allelic variants of the same antigen (AMA1) using a multiplex assay was assessed by comparing AMA1-specific antibodies levels (expressed as MFI) from such multiplex assays with levels against the same alleles using singleplex assays. A subset of 42 plasma samples from one of the sampling time points was selected for this comparative analysis. Fifty microlitres (50 μl) of the antigen-coupled bead mixture were distributed into microtitre wells and 50 μl of 1:400 diluted plasma samples added to duplicate wells in the plate. For the multiplexed assay, equal numbers of the four AMA 1 variant coupled beads were mixed together and used to determine antigen-specific antibody levels in the selected plasma samples. Each AMA1 variant coupled bead was concurrently run as a singleplex using the same diluted plasma samples for comparison, and beads coupled with BSA were included in both multiplex and singleplex assays as a negative control. The median MFI of data from singleplex and multiplex assays were subsequently compared.

### Statistical analysis

Non-parametric analyses were performed since the antibody data were not normally distributed. Differences in median antibody MFI between singleplex and multiplex assays for each AMA1 allelic variant were assessed by Mann-Whitney U tests. For each antigen, the multiplex and singleplex MFI data were also subjected to Spearman rank correlation analysis.

At each of the six sampling time points, the median antigen-specific antibody levels were compared between the two study groups. Antigen-specific antibody levels were also compared across the six sampling time points by Kruskal-Wallis test followed by the Bonferroni pair-wise post-hoc test.

For assessment of the proportions of cross-reactive and strain-specific antibodies to the four allelic variants of AMA1, antibody data was log-transformed to the base 2 and subjected to Bland-Altman analysis using the R package *ggplot2* (R script and data file provided as S1 Appendix and S2 Appendix, respectively). The geometric mean of differences between the paired antigen-specific antibodies is displayed as a bold horizontal line, known as the line of equality, and the 95% limits of agreement for the paired data distribution are displayed by dotted horizontal lines. Ideally, if paired AMA1 allelic variants recognize antibodies in plasma samples to the same extent, their plotted data points will be on the line of equality. If paired AMA1 variants do not recognize antibodies in plasma samples to the same extent, however, the plotted data points will be further away from the line of equality.

For the assessment of potential antibody correlates of protection against clinical malaria, a generalized mixed effects regression model for repeated measurements was fitted to antigen-specific antibody data using the R package *lme4* [34]. Initially, antigen-specific antibody data were entered into the model as fixed effects with age as an interaction term, while sampling time point and study subject were entered as random effects. This model was compared to other models that either dropped the subject age interaction term or one of the random effects, and the best model, based on the lowest AIC values was selected for further analysis. The best model had log_2_–transformed antibody level as the fixed effect and study subject as a random effect since the interaction term (age) and sampling time point did not significantly contribute to the models. P-values for the intercept and fixed effect were obtained by likelihood ratio tests (with Laplace Approximation). All analyses and graphics were done using either the R statistical software (Version 3.4.0) or the GraphPad Prism software (version 5, San Diego, USA) and differences were considered to be statistically significant when p values were less than 0.05.

## Results

### Study participant groups

**Fig 1:**
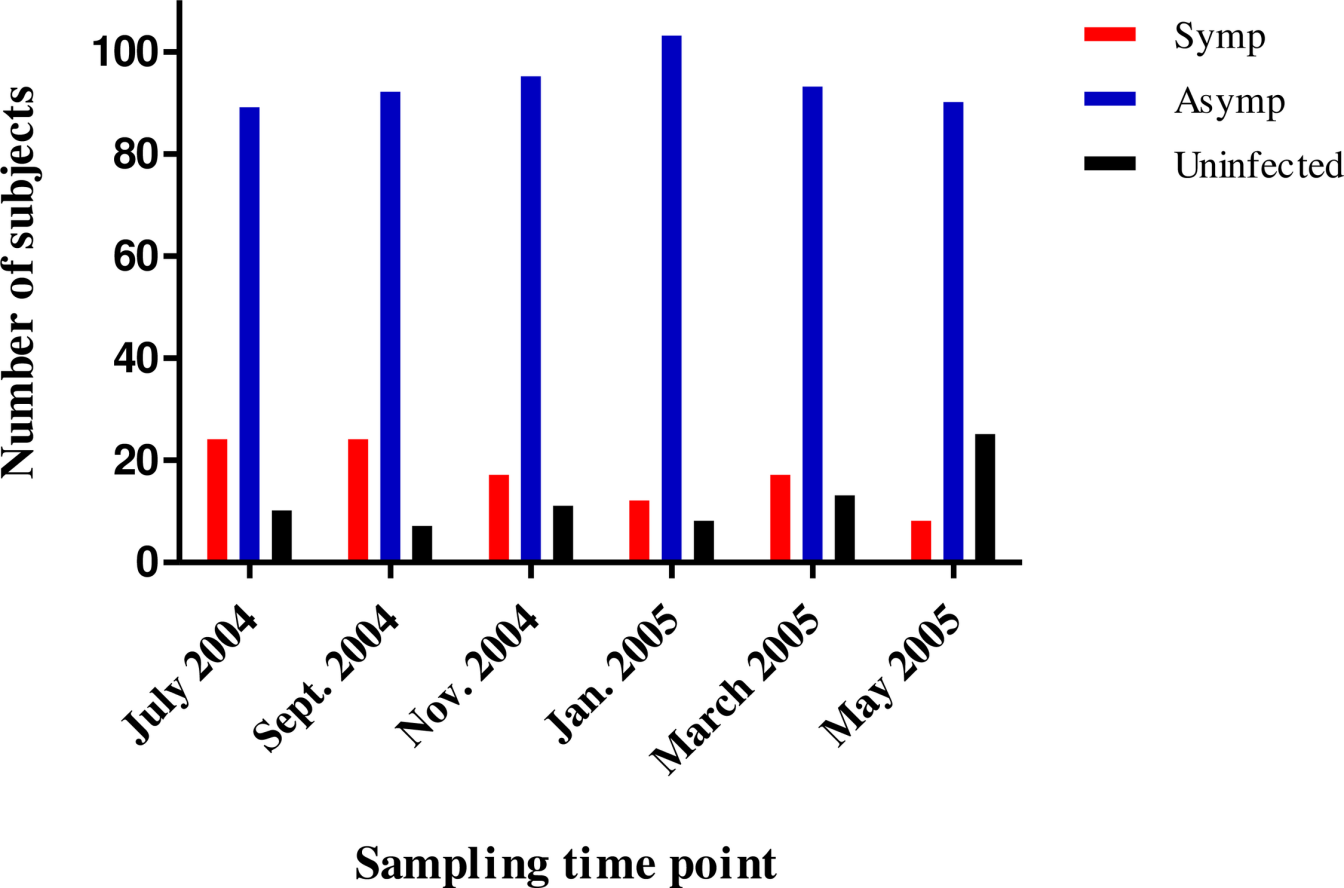
Numbers of symptomatic and asymptomatic children at the six sampling time points. At each sampling time point (from July 2004 to May 2005), the 126 children were grouped into three categories; children with clinical malaria (blood film parasitaemia, fever and at least one other symptom of malaria and no other obvious cause for the fever, described as Symp), children with blood film parasitaemia but no clinical symptoms (Asymp) and children with no blood film parasites (Uninfected).

Plasma samples collected from 126 children at six different time points (total sample size 756) were analyzed in this study. This number included 64 children who had clinical malaria symptoms at least once over the six sampling time points and 62 children who had high asymptomatic parasitaemia at least once over the six time points. There were 108 clinical malaria episodes over the entire study period; 25 clinical episodes were recorded in July 2004 and another 25 in September 2004, 18 episodes were recorded in November 2004, 13 episodes in January 2005, 18 episodes in March 2005 and 9 episodes in May 2005 (Fig 1). Overall, 43 of the 64 symptomatic children experienced a single clinical episode, eight children had two episodes, seven children had three episodes while three children had four clinical episodes over the entire study period. Two children had five episodes and a single child had clinical episodes at all six time points. The proportion of symptomatic children was generally lower compared to that of asymptomatic children at all time points, and these two groups differed in study subject composition from one sampling time point to another. Only a few of the 126 children had no blood film parasites at any sampling time point, and the proportion of these children showed a trend of increase towards the dry season (Fig 1). There were no significant differences in parasite density between symptomatic and asymptomatic children at all time points.

### Comparison of multiplex and singleplex assays

An initial assessment of the capacity of multiplex assays to accurately estimate the levels of specific antibodies to allelic variants of the same antigen was performed by measuring and comparing antibody levels in selected samples by singleplex and multiplex assays. Median MFI levels were not significantly different between singleplex and multiplex assays for any of the four allelic AMA1 variants (P > 0.05 in all cases, Mann-Whitney test, Table 1). Spearman correlation analysis of data also showed that MFI data from singleplex and multiplex assays were significantly correlated for all the antigens (Table 2). Though the correlation between FVO AMA1 levels measured by singleplex and multiplex assays was comparatively weaker (r = 0.4634), those for the other three allelic variants were much stronger, with correlation coefficients being greater than 0.8 (Table 2). Thus measurement of plasma antibody levels against the four allelic AMA1 antigens using multiplex assays generally yielded similar results as measurements of the same allele-specific antibody levels in singleplex assays.

**Table 1:** Comparison of singleplex and multiplex assays for measurement of AMA1 allele-specific antibody levels.

Antigen	Number of samples	Median level (MFI)		P-value (two-tailed)
		multiplex	singleplex	
3D7	42	2254 (14–9366)	2922 (18–8305)	0.60
FVO	41	1211 (18–5818)	1418 (11–4777)	0.59
HB3	42	2263 (24–8097)	1697 (27–5653)	0.07
CAMP	41	2246 (153–7568)	2187 (22–5611)	0.36

Values reported as median fluorescence intensity (MFI) level (min–max)

**Table 2:** Correlation between singleplex and multiplex assays for AMA1 allele-specific antibodies.

Antigen	Number of sample pairs	Spearman r	P value (two-tailed)
3D7 AMA1	42	0.9421	< 0.0001
FVO AMA1	41	0.4634	0.0023
HB3 AMA1	42	0.9056	< 0.0001
CAMP AMA1	41	0.8481	< 0.0001

### Comparison of malaria antigen-specific antibody levels amongst study groups and across sampling time points

**Fig 2:**
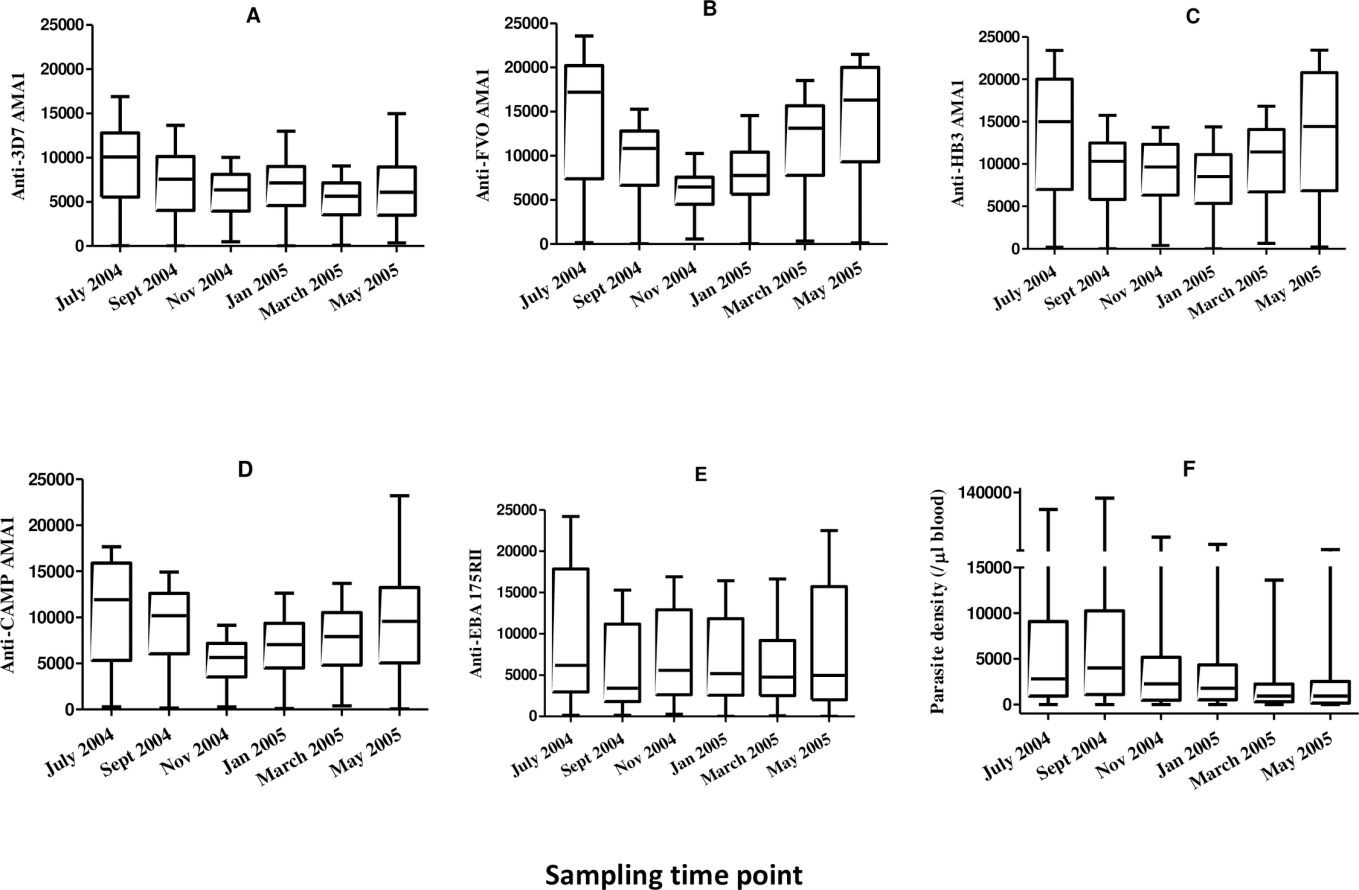
Antigen-specific antibody level and parasite density variations over the study period. Levels of either the antigen specific antibodies (A-E) or parasite density (F) at the six sampling time points were compared by the Kruskal-Wallis test, followed by the Bonferroni post-hoc test to assess pair-wise differences. Results (p values) after post-hoc tests are presented in S2 Table.

Antibodies in plasma samples from the six sampling time points were measured against EBA 175RII and the four allelic variants of AMA1 (from the 3D7, FVO, HB3 and CAMP parasite strains). For all antigens, there were no significant differences in median antigen-specific antibody levels (MFI) between the two study groups at any of the six time points (S1 Table). Comparison of antigen-specific antibody levels across the six sampling time points however showed that antibody levels against the four AMA1 variants generally showed a trend of decline from July 2004 and were lowest in November 2004/January 2005, and this trend was more pronounced for anti-FVO and anti-CAMP AMA1 antibody levels (Fig 2A–2D, S2 Table). For two of the antigens (FVO and HB3), anti-AMA1 levels subsequently showed a steady increase from January 2005 through March 2005 to May 2005 when levels became comparable to those in July 2004 (Fig 2B and 2C, S2 Table). Anti-EBA 175RII antibody levels were however generally maintained across the six time points (Fig 2E, S2 Table). Similar trends were observed when anti-specific antibody levels of either symptomatic children alone or asymptomatic children alone were compared across the six time points. Thus anti-AMA1 antibody levels showed a trend of increase during the rainy season and a trend of decrease during the dry season, as is expected. Anti-EBA 175RII levels however were generally more stable over the study period.

Although parasite density was not significantly different between symptomatic and asymptomatic children at any of the six sampling time points (S1 Table), parasite density varied significantly across the time points (Fig 2F, S2 Table). Three clusters of parasite levels could be clearly identified from the data; the highest were seen in July and September 2004, which also coincide with the peak transmission period. A statistically significant decline from the September 2004 levels to intermediate levels was then seen in November 2004 and January 2005 (Fig 2F, S2 Table). Parasite density in March and May 2005 were the lowest, and were statistically significantly different from parasite densities in November 2004 and January 2005.

### Assessment of relative proportions of cross-reactive and strain-specific anti-AMA1 antibodies

**Fig 3:**
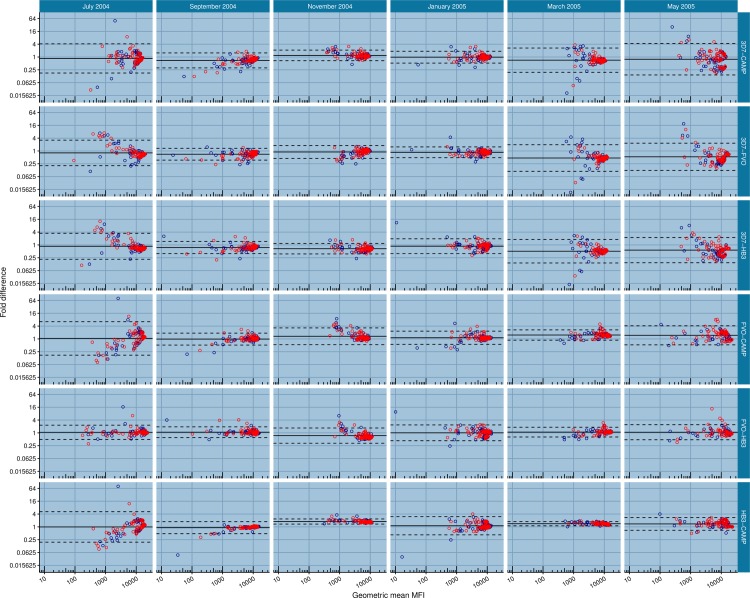
Pair-wise comparison of AMA1 allele-specific antibody levels. Points in a panel represent a plot of the fold difference between paired antibody levels (for example, the 3D7-CAMP panel plots the log-transformed anti-3D7 antibody levels minus anti-CAMP antibody levels) in plasma samples against the geometric mean (antilog) of the same paired allele-specific antibody levels. The bold horizontal line (line of equality) in each panel represents the average of all the differences between antibody levels against the specified antigen pair. The dotted horizontal lines represent the 95% limits of agreement for the distribution. Red open circles represent data from children with symptomatic malaria (Symp) and blue open circles are data from children with asymptomatic infections (Asymp).

Plasma antibody levels against the four variants of AMA1 were log transformed (base 2) and the levels compared in a pair-wise manner using Bland-Altman plots (Fig 3). For each pair of AMA1 allelic variants, this was to evaluate the extent of agreement between antibodies levels against one AMA1 variant with those against another variant. Generally, narrow 95% limits of agreement suggest a high level of agreement between paired allele-specific antibody levels, and this is indicative of a higher proportion of antibodies recognizing epitopes that are shared by the two AMA1 allelic variants (cross-reactive antibodies). Conversely, wider 95% limits of agreement suggest a lower level of agreement and imply the recognition of a significant proportion of unique epitopes by one antigen variant relative to the other (strain-specific antibodies).

Overall, the narrowest 95% limits were observed for plots between the HB3/CAMP paired variants of AMA1 in November 2004 and March 2005 (Fig 3), suggesting that a high proportion of anti-AMA1 antibodies in samples taken at these time points recognized epitopes that are shared by these two AMA1 variants. The widest 95% limits were observed for plots between the FVO/CAMP and HB3/CAMP paired variants in July 2004 as well as for 3D7/CAMP paired variants in May 2005 (Fig 3), indicating that a higher proportion of anti-AMA1 antibodies during that period of the year mostly recognized antibody epitopes that are unique to certain AMA1 allele variants.

For pair-wise comparisons that included antibodies against the 3D7 AMA1 variant, the 95% limits observed in July 2004, March 2005 and May 2005 were at least two-fold wider compared to the corresponding 95% limits in September 2004, November 2004 and January 2005. These collectively suggest the predominance of a more cross-reactive anti-AMA1 antibody profile during periods of moderate to high disease transmission (at peak and end of the rainfall period) and an increase in the levels of strain-specific anti-AMA1 antibodies during the dry season and before the next major transmission season begin.

**Fig 4:**
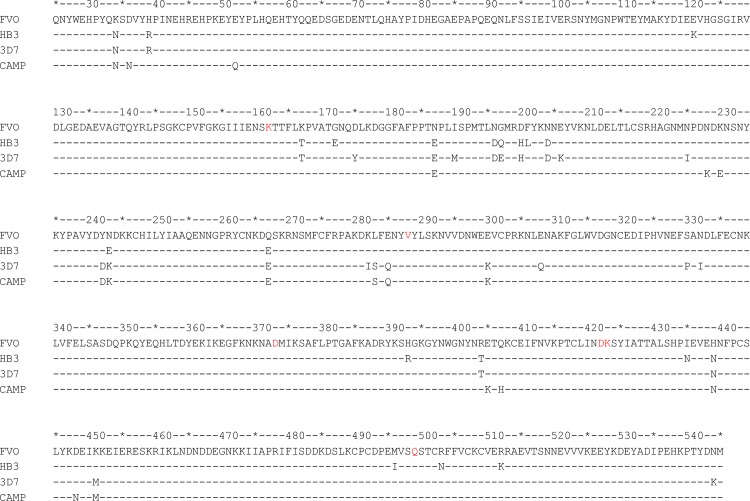
Comparison of protein sequences (aa25–545) of the for AMA1 antigen variants. All antigens were produced in *Pichia pastoris* and devoid of N-glycosylation sites. These have been replaced with amino acid residues (indicated in red) that occur in AMA1 sequences from other *Plasmodium* parasites (N162K, T288V, S373D, N422D, S423K, N499Q). Each protein consists of a portion of the prodomain (aa25–96), domain I (aa97–315), domain II (aa316–425) and domain III (aa426–545). All antigens reacted with the reduction-sensitive rat monoclonal antibody 4G2 on western blots (Faber et al 2008), which was taken as a surrogate measure of conformational integrity.

The extent of antibody cross-reactivity between any two AMA1 variants may be directly related to the degree of sequence similarity between the antigen vaiants. Alignment of the amino acid sequences of the four AMA1 variants used in this study (Fig 4) shows that the FVO and HB3 AMA1 variants differ by a total of 20 amino acids (2 in the prodomain, 11 in domain I, 2 in domain II and 5 in domain III) while the FVO and 3D7 variants differ by 26 amino acids (2 in the prodomain, 18 in domain I, 3 each in domains II and III). The FVO and CAMP variants differ by 17 amino acids (3 in the prodomain, 9 in domain I, 2 in domain II and 3 in domain III), HB3 and 3D7 variants differ by 24 amino acids (15 in domain I, 3 in domain II and 6 in domain III), HB3 and CAMP variants differ by 29 amino acids (3 in the prodomain, 16 in domain I, 4 in domain II and 6 in domain III) and the 3D7 and CAMP variants differ by 23 amino acids (3 in the prodomain, 13 in domain I, 5 in domain II and 2 in domain III). Thus the FVO and CAMP AMA1 variants have the greatest sequence similarity while the HB3 and CAMP variants have the least sequence similarity (Fig 4).

On the whole, there was no clear trend of differences between symptomatic and asymptomatic children with respect to the relative proportions of cross-reactive and strain-specific antibodies across all six sampling time points.

### Malaria-specific antibodies and the risk of clinical malaria

To identify potential antibody correlates of protection against malaria, a mixed effect linear regression model was fitted for each antigen with clinical malaria status as the binary outcome variable, antigen-specific antibody data over the six time points entered as fixed effects and study subject as a random effect. Data from the asymptomatic study group were used as the reference for comparison with data from individuals who suffered at least one clinical malaria episode over the six time points. For all antigens (EBA 175RII and the four AMA1 variants), there was no association between measured antibody levels and clinical malaria status (Table 3). However, in some cases, the statistically significant difference between intercepts indicates that there are additional factors not considered in this analysis that may account for malaria outcome in study subjects.

**Table 3:** Relation between antibody levels and clinical malaria outcome^#^.

Antigen	Fixed effects	Estimate	Standard error	P value (two-tailed)
**3D7 AMA1**	Intercept	12.198	6.160	0.051
	Log_2_(Ab level)	0.046	0.490	0.927
**FVO AMA1**	Intercept	12.488	6.206	0.044
	Log_2_ (Ab level)	0.021	0.474	0.964
**HB3 AMA1**	Intercept	12.887	0.0009	< 0.0001
	Log_2_ (Ab level)	-0.001	0.0009	0.414
**CAMP AMA1**	Intercept	12.822	6.702	0.056
	Log_2_ (Ab level)	-0.005	0.524	0.993
**EBA 175RII**	Intercept	12.649	5.097	0.013
	Log_2_ (Ab level)	0.009	0.408	0.981

A mixed effects logistic model was fitted to the repeated antibody data measurements to predict the risk of clinical malaria, with study subject treated as a random effect variable.

^#^The model assessed clinical malaria risk with the asymptomatic infection group as reference. Age as an interaction term did not significantly impact the model, most likely due to the narrow age range, and it was dropped from the model.

## Discussion

The acquisition of malaria-specific antibodies is very important for the establishment of partial clinical immunity to malaria since antibodies play a crucial role in parasite clearance and a reversal of clinical disease symptoms [3,5]. Though the exact targets of protective immune responses have not been completely defined, a number of parasite antigens from multiple life cycle stages have been identified as potential targets [7,8,35,36]. Although most of these antigens are polymorphic and would therefore elicit a mix of strains-specific and cross-reactive responses, the importance of antigen polymorphisms on the functionality and levels of elicited immune responses has not been given much consideration. The gradual development of clinical immunity to malaria with age after repeated exposure to diverse parasites has either been associated with the induction of a diverse repertoire of strain-specific immune responses or with the induction of a predominantly cross-reactive (strain-transcending) immune response profile [2]. Some studies have shown that children generally elicit antibody responses with a greater proportion of strain specificity as compared to adults, who develop a more cross-reactive immune response by virtue of their long periods of exposure to diverse parasite strains [22,23,37]. It has also been demonstrated in animal models that immunization with a mixture of allelic antigen variants focuses the antibody response on epitopes that are common amongst the variants [26,27]. Taken together, it can be hypothesized that individuals who experience simultaneous infection with multiple parasite variants will develop a more cross-reactive antibody profile compared to those who mostly experience clonal infections. A number of studies have demonstrated a greater average number of parasite variants per infected person, expressed as the multiplicity of infection (MOI), under high malaria transmission settings compared to corresponding findings in areas of low transmission [38–40]. The current study therefore assessed the functional quality of antibody responses in symptomatic and asymptomatic children between the ages of one and five years from an area in the Upper-East Region of Ghana with clearly marked seasonal malaria transmission. Antibody levels against four allelic variants of the blood stage antigen AMA1 as well as those against a single variant of EBA 175RII were measured by multiplex assays and compared amongst symptomatic and asymptomatic children who were sampled every two months over a period of one year. The usefulness and reliability of multiplex assays for determining the levels of various analytes in complex fluids such as plasma, serum and culture supernatants has been amply demonstrated [41–45]. To assess the feasibility of measuring the levels of antibodies to allelic variants of the same antigen in a multiplexed manner, we initially performed and compared multiplex and singleplex measurements of antibody levels against four allelic AMA1 variants using plasma samples from a subset of the study population. Data from these initial tests showed that AMA1 allelic-specific antibody levels could be measured in a multiplexed manner and were similar to the levels obtained in variant-specific singleplex assays that were run concurrently (Tables 1 and 2). This therefore confirms previous reports of the feasibility of measuring antibody levels against allelic variants of the same antigen in multiplex assays [46].

The measured antigen-specific antibody levels for all study subjects were compared across the six sampling time points. Antibody levels against variant AMA1 allele types were generally higher during the high transmission season compared to the low transmission season, while anti-EBA 175RII levels did not change considerably between seasons (Fig 2A–2E, S2 Table). The measured antigen-specific antibody levels were however not significantly different between clinically ill and asymptomatically infected children at any of the six sampling time points (S1 Table). These collectively suggest that anti-AMA1 antibodies may merely be markers of exposure to parasites in this age group since the antibody levels change with transmission intensity, which is closely linked with rainfall patterns [31,47,48]. A similar observation of higher rainy season anti-AMA1 antibody levels has been made previously in this age group (1–5 years) within the same study district [49]. In this other study, anti-AMA1 antibody levels in older children (> 5 years) and adults were not significantly different between the rainy and dry seasons.

Parasite density was high in July 2004 and peaked in September 2004, beyond which there was a decline to intermediate density in November 2004 and January 2005 (Fig 2F, S2 Table). Parasite density was at the lowest in March and May 2005, and this pattern of changes in parasite density over a complete transmission year have been previously described [31,47]. This pattern clearly confirms the slight offset of the rainy (May to October) and dry (November to April) seasons from the high and low malaria transmission periods in the study area.

The lack of statistically significant differences in antibody levels between symptomatic and asymptomatic children at all time points corroborate the outcome of the mixed effects logistic models, that none of the measured antibodies showed an association with clinical malaria outcome (Table 3). Baseline data from the original study has been published previously and show an association between anti-3D7 AMA1 antibody levels and a reduced risk of clinical malaria over the study period [30]. While this previous study utilized samples at a single time point from 325 study subjects [30], the current study analyzed six follow up samples from a subset of 126 subjects. Clinical malaria risk analysis in the previous study included asymptomatic children with and without blood film parasitaemia and a clinical malaria definition that had a parasitaemia cut-off of > 5000 parasites per μl of blood, while the current study excluded subjects with no blood film parasitaemia and used a clinical malaria definition that included any level of parasitaemia. The observed differences in outcomes between the two studies could therefore be attributed to these definition and classification differences. It is worth noting, however, that while some epidemiologic studies have associated levels of specific antibodies to these two antigens with a reduced risk of clinical malaria [6,50–53], others have failed to confirm these associations [48,54,55].

For assessment of the relative proportions of strain-specific and cross reactive antibodies, Bland-Altman plots revealed a relatively higher preponderance of cross-reactive antibodies from September 2004 to January 2005, which cover the peak rainfall season and into the early part of the dry season, while relatively higher levels of strain-specific antibodies were elicited during the low transmission season and into the early part of the rainy season (Fig 3). This observation may be closely linked with the diversity of infecting parasite strains over the transmission period; high transmission periods have been associated with multiple parasite strain infections, reflected by high MOIs in infected individuals while low transmission periods are predominantly associated with a greater proportion of clonal parasite infections [38–40,56–58]. These patterns are most likely a direct result of an upsurge in transmission vector population during the rainy periods and a decline during the dry periods [31,47]. Concurrent infection with multiple parasites is likely to focus the antibody response on epitopes that are shared by the infecting parasite variants, while clonal infections would most likely focus responses on targets within the single allele variants, which include both cross-reactive and strain specific epitopes [22,23]. Thus during periods of low malaria transmission, relatively high proportions of strain-specific antibody responses are likely to be elicited due to the reduced number of circulating parasite strains per infected individual.

Though the parasite exposure history of the study population is not known, the pattern of antibody responses to the four AMA1 variants suggests possible exposure to a diversity of parasite variants over the one year follow up period. The observation of narrow limits of agreement during the high transmission period and broad limits of agreement during the low transmission period is consistent for antigen pairs that include 3D7 AMA1 but not paired comparisons involving any of the other three variants (FVO/CAMP, FVO/HB3 and HB3/CAMP, Fig 3). The extent of antibody cross-recognition by the four AMA1 variants is dependent on how similar these antigens are in sequence and structure. The 3D7 AMA1 variant differs by between 23 to 26 amino acids from the other three AMA1 variants (Fig 4), including residue 197 which is the most polymorphic position and has been described as part of an important immunodominant epitope in domain I of AMA1 [26,59].

The HB3 and CAMP AMA1 antigens show the least sequence identity amongst the four antigens (Fig 4), and the observation of the narrowest limits of agreement between these two antigens variants over most of the sampling time points would suggest two things; i) that parasites circulating in the study area may have AMA1 sequences that differ significantly from these two variants, and ii) that the HB3 and CAMP variants are binding to the fraction of infection-induced antibodies that recognize common, cross-reactive epitopes.

A limitation of the current study was our inability to directly estimate MOIs based on AMA1 variants at the sampling time points due to the unavailability of the appropriate corresponding samples. This would have provided direct evidence of the association between MOI and the quality of antibody responses. This notwithstanding, the emerging pattern of association between MOI, albeit based on mostly MSP2 allele families, and transmission intensity in malaria endemic areas [38–40,56–58] provides the necessary basis for making the inferences drawn in this study.

## Conclusions

This study confirms a previous report that multiplexed Luminex assays are as effective as singleplex Luminex assays for the measurement of antibody levels against allelic forms of the same antigen. Anti-AMA1 antibodies may be potential markers of exposure and could be especially useful in showing differences between high and low transmission areas. Despite the lack of association of any antigen or variant-specific antibody responses with protection from clinical malaria, the data shows a possible relationship between transmission intensity and the specificity of antibody responses. Cross-reactive antibodies were predominantly present during periods of high transmission, most likely due to higher MOI which is believed to be characteristic of the high malaria transmission. In contrast, an increased proportion of strain-specific antibodies was present during periods of low malaria transmission, most likely as a result of a greater preponderance of clonal infections. The data therefore adds to our current understanding of the acquisition of antibody specificities in relation to transmission intensity and infecting parasite diversity.

## Supporting information

S1 Table Comparison of antigen-specific antibody levels and parasite between the two study groups for each sampling time point. Comparison of antigen-specific antibody levels amongst the two study groups for each sampling time point. Antibody level data is presented as the Median MFI (min.—max.)**. *Symp*** refers to clinically symptomatic children with any level of parasitaemia, fever and at least one other symptom of malaria. ***Asymp*** refers children with no clinical symptoms but detectable parasites in blood smear. P values obtained after Mann-Whitney U analysis.
(XLSX)

S2 Table Results of pair-wise post-hoc tests for anti-AMA1 antibodies and parasite density across the six sampling time points. Results of pair-wise post-hoc tests for anti-AMA1 antibodies across the six sampling time points. Bonferroni post-hoc test was used to assess pair-wise differences in antibody levels across the different sampling time points.
(XLSX)

S1 Appendix R script for construction of the multi-level Bland-Altman plots. (TXT)

S2 Appendix Data file for Bland-Altman plots. (CSV)

S3 Appendix R script and data output used for regression model selection. (TXT)
